# Conducting evaluations of evidence that are transparent, timely and can lead to health-protective actions

**DOI:** 10.1186/s12940-022-00926-z

**Published:** 2022-12-05

**Authors:** Nicholas Chartres, Jennifer B. Sass, David Gee, Simona A. Bălan, Linda Birnbaum, Vincent James Cogliano, Courtney Cooper, Kristi Pullen Fedinick, Roy M. Harrison, Marike Kolossa-Gehring, Daniele Mandrioli, Mark A. Mitchell, Susan L. Norris, Christopher J. Portier, Kurt Straif, Theo Vermeire

**Affiliations:** 1 Program On Reproductive Health and the Environment, Department of Obstetrics, Gynecology, and Reproductive Sciences, University of California at San Francisco, 490 Illinois Street, Floor 10, San Francisco, CA 94143 USA; 2 Natural Resources Defense Council, Washington, DC USA; 3 George Washington University, Washington, DC USA; 4 Brunel University, London, UK; 5 California Department of Toxic Substances Control, Berkeley, CA USA; 6 University of California at Berkeley, Berkeley, CA USA; 7 Nicholas School of the Environment, Duke University, Durham, NC USA; 8 California Office of Environmental Health Hazard Assessment, Oakland, CA USA; 9 School of Geography, Earth & Environmental Sciences, University of Birmingham, Birmingham, UK; 10 Department of Environmental Sciences/Centre of Excellence in Environmental Studies, King Abdulaziz University, Jeddah, Saudi Arabia; 11 Department of Environmental Hygiene, Section Toxicology, Health Related Environmental Monitoring, German Federal Environmental Agency, Dessau-Roßlau, Germany; 12 Cesare Maltoni Cancer Research Center, Ramazzini Institute, Bologna, Italy; 13 George Mason University, Fairfax, VA USA; 14 Connecticut Coalition for Environmental Justice, Hartford, CT USA; 15 Department of Family Medicine, Oregon Health & Science University, Portland, OR USA; 16 Rollins School of Public Health, Emory University, Atlanta, GA USA; 17 Department of Toxicogenomics, Maastricht University, Maastricht, Netherlands; 18 CJP Consulting, Seattle, WA USA; 19 ISGlobal, Barcelona, Spain; 20 Boston College, Newton, MA USA; 21 Retired, National Institute for Public Health and the Environment (RIVM), Utrecht, The Netherlands

**Keywords:** Conflicts of interest, Industry sponsorship, Environmental justice, Cumulative impacts, Non-chemical stressors, Precautionary principle, Risk of bias, Systematic review, Transparency

## Abstract

**Background:**

In February 2021, over one hundred scientists and policy experts participated in a web-based Workshop to discuss the ways that divergent evaluations of evidence and scientific uncertainties are used to delay timely protection of human health and the environment from exposures to hazardous agents. The Workshop arose from a previous workshop organized by the European Environment Agency (EEA) in 2008 and which also drew on case studies from the EEA reports on ‘Late Lessons from Early Warnings’ (2001, 2013). These reports documented dozens of hazardous agents including many chemicals, for which risk reduction measures were delayed for decades after scientists and others had issued early and later warnings about the harm likely to be caused by those agents.

**Results:**

Workshop participants used recent case studies including Perfluorooctanoic acid (PFOA), Extremely Low Frequency – Electrical Magnetic Fields (ELF-EMF fields), glyphosate, and Bisphenol A (BPA) to explore myriad reasons for divergent outcomes of evaluations, which has led to delayed and inadequate protection of the public’s health. Strategies to overcome these barriers must, therefore, at a minimum include approaches that 1) Make better use of existing data and information, 2) Ensure timeliness, 3) Increase transparency, consistency and minimize bias in evidence evaluations, and 4) Minimize the influence of financial conflicts of interest.

**Conclusion:**

The recommendations should enhance the production of “actionable evidence,” that is, reliable evaluations of the scientific evidence to support timely actions to protect health and environments from exposures to hazardous agents. The recommendations are applicable to policy and regulatory settings at the local, state, federal and international levels.

## Introduction

In February 2021, approximately one hundred scientists and policy experts participated in a workshop, *Conducting Evaluations of Evidence that are Transparent, Timely and Lead to Health-Protective Actions,* convened and co-hosted by United States (U.S.) and United Kingdom (UK) academic institutions and public interest groups to discuss barriers to timely actions that could protect public health and the environment from unsafe exposures to hazardous agents [1]. The Workshop arose from a previous workshop organized by the European Environment Agency (EEA) in 2008 and drew on case studies from the EEA reports on *Late Lessons from Early Warnings* [2, 3]. These two reports documented dozens of hazardous agents including many chemicals, for which risk reduction measures were delayed for decades after scientists and others had issued early and later warnings about the harm likely to be caused by those agents.

Overwhelmingly, when it comes to human suffering from chemical exposures, “Environmental Justice Communities,” or “EJ Communities,” whose residents are predominantly of color, and/or low income, are disproportionately impacted [4–6]. These communities are burdened by the cumulative effects of multiple hazardous industries sited closely together, in addition to other chemical stressors from the products they use in their homes and non-chemical stressors such as poverty, racial discrimination, and poor access to regular affordable medical care [7]. This structural racism and classism, described by Donley et al. (2022) as systems that result from historical, institutional, cultural or behavioral societal actions that disadvantage and harm low-income and communities of color [6], contribute to persistent environmental health disparities in these populations [4, 8]. In the U.S., over 130 million people live in the vulnerability zones surrounding 3,433 facilities that produce, store, and use highly hazardous chemicals, as identified by the U.S. Environmental Protection Agency (EPA) Risk Management Planning program [9]. Residents of these vulnerable zones are overwhelmingly Black and Latino, with higher rates of poverty than the general U.S. population. These disparities are even greater in the “fenceline zone” within roughly one mile around an industrial facility [9, 10]. Governments and others have repeatedly failed these communities, even with the most basic protections afforded under the law [1, 11].

With so many examples of harm to people and the environment from delayed health and environmental protection policies and practices, why are early warnings from scientists still not sufficient to spur rapid action to ban production, replace hazardous materials with safer alternative products and processes, install pollution controls, and take other measures to avoid harm? The situation is made much more complex for myriad reasons, including but not limited to, the chemical industry’s influence in the regulatory process, the expense of conducting toxicity testing and environmental monitoring of chemicals, the complexities posed by chemical mixtures and how to determine the toxicity of an individual chemical, and ever-changing product formulations, the lack of public disclosure of both hazard and exposure information, the reality of multiple-chemical exposures (both aggregate exposure of an individual chemical from multiple sources, and cumulative risk from multiple chemicals) that is higher for workers and many communities, particularly low income and communities of color, and manufacturer’s claims of economic hardship due to the costs of installing pollution controls and other safety measures [5, 12]. Solutions will need to find the appropriate balance between the health risks and social benefits of chemicals and encourage safe, sustainable business strategies that are informed by communities. Importantly, risk management analyses, including benefit–cost analyses, should not only consider the aggregate health benefits to the whole exposed population but also the distribution of those benefits to the most impacted and vulnerable communities, defined by race/ethnicity and socioeconomic status indicators, including but not limited to educational attainment, income, and immigration status. These analyses must explicitly quantify baseline risks to each vulnerable population in the current scenario, the expected risks for each vulnerable population after implementation of a regulatory decision, and the resulting risk reduction for each vulnerable population. As the 1998 report on the State of Europe’s Environment warned, “Each year that passes without effective action will result in decades of additional, unintended exposure to chemicals that are likely to be harmful to human health and the environment” [13].

## Methods

During an online workshop, held over four days in February 2021, participants discussed recent case studies with the goals of a) identifying cross-cutting barriers to protective actions; b) suggesting strategies to overcome these barriers; and c) making recommendations to overcome key barriers.

Over the four days, speakers and participants discussed the social impacts of delayed health protections, identified some of the barriers to conducting transparent and timely evaluations, and proposed solutions for communicating uncertainty and translating scientific evidence of harm into health-protective actions. The Workshop offered a productive space to identify common hurdles that hinder health protection, as well as best practices for moving forward with the ongoing work to protect people around the world from exposure to hazardous materials.

A Proceedings of the Workshop is publicly available from the University of California, San Francisco’s Program on Reproductive Health and the Environment (UCSF-PRHE) website [1]. It includes the workshop agenda, speakers’ affiliations and biographies, speakers’ PowerPoint slides, and short written summaries of each day’s small-group meetings discussions. This paper comes out of the presentations and discussions of the workshop, but is not a reporting of the workshop, which was done in the Proceedings.

## Results

### Cross-cutting barriers to protective action

Some prominent examples of divergent evaluations from environmental and public health that were discussed and analyzed during the 2021 workshop and the EEA 2008 workshops included bisphenol A (BPA), pesticides spray drift, hexavalent chromium, glyphosate, nitrogen dioxide, perfluorooctanoic acid (PFOA), fluoride, endocrine disrupting substances [14], and extremely low frequency electromagnetic field (ELF-EMF) radiations from power lines and from mobile telecommunications. Such case studies revealed many of the reasons why there were divergent evaluations between different risk assessments, such as those between the International Agency for Research on Cancer (IARC) and European Union (EU) Chemical and Food Agencies over glyphosate; and between four risk assessments on PFOA that produced very different recommendations on protective exposure limits.

More rarely there are divergent opinions on the appropriate methods to evaluate the science, and thus reach different conclusions from within the same committee, as with the UK report on nitrogen dioxide which was explicit about the reasons for such divergent views [15]. These included disagreements over: the appropriate use and interpretation of models for multi-pollutant exposures; extrapolations beyond studied concentrations; appropriate strengths of evidence needed to support likely causal associations compared to that needed to support a reliable estimate of the quantitative effects of exposure on health impacts and health benefits; and the consideration of uncertainties in the evidence. In a subsequent systematic review of the evidence as part of the process of recommending air quality guidelines, the World Health Organization (WHO) acknowledged the greater weight of evidence favoring causality in the UK report, and that new evidence was also consistent with this view [16]. There is often much less transparency about the causes of divergent evaluations by different committees of the same evidence, but analyses of the case studies presented to the workshops revealed many of them. While additional case studies were presented and discussed at the 2021 Workshop, we briefly present four exemplary cases below.

#### Example of PFOA

Four different risk assessments of PFOA conducted by the Dutch National Institute for Public Health and the Environment (RIVM, 2016), US EPA (2016), the European Food and Safety Authority (EFSA, 2018), and the German Umweltbundesamt (UBA, 2018), resulted in four different Health Based Guidance as follows: RIVM, 89 ng/mL serum, based on liver effects from animal studies together with safety factors; US EPA, 30 ng/mL serum based on animal studies with safety factors; EFSA, 9 ng/mL serum based on epidemiologic studies with elevated serum cholesterol as the critical effect; and, UBA, 2 ng/mL serum, based on epidemiology [1]. These were later analyzed by RIVM and Utrecht University [17], which identified the lack of documentation on the underlying motives and preferences for using either epidemiology or toxicology and on the selection of relevant endpoints. Aspects considered in the argumentation analysis were sources of evidence used and reasons to include or exclude evidence, what key evidence weakened or strengthened the weight of evidence on causality, what ancillary evidence was used and how weight of evidence was phrased or categorized. The authors recommended more in-depth analysis and transparency of the chain of argumentation is needed to better disclose the underlying reasoning leading to the choice of the critical study or studies and critical endpoints, and in this case whether toxicology or epidemiologic data was selected as the primary support for the guidance values. The study authors note that more explicit identification and discussion of initial beliefs, assumptions and starting points for the argumentation could be a valuable addition to general risk assessment frameworks to make maximum use of both the toxicological and epidemiological data and expedite shared conclusions. Clear and transparent documentation and reasoning is necessary for communicating the underlying argumentation, and is important to enhance public and policymaker understanding of the different beliefs, assumptions, choices, and judgements that help produce such divergent evaluations of evidence [1, 17].

#### Example of ELF-EMF fields

Even where human evidence alone is used in the evaluations, the case study on ELF-EMF radiation showed that there is a risk that review bodies may overlook evidence of adverse effects in a collection of disparate studies that are individually inconclusive [18]. Common sources of ELF-EMF are power lines, electrical wiring, and electric appliances. Whilst IARC (2002) [19] based its “possible” human carcinogenicity (Group 2B) determination on only one cancer endpoint, childhood leukemia, a review of the same human evidence by the California Department of Health Services (CDHS) in 2002 [20] identified three cancer outcomes as each warranting a “possible” carcinogen classification: childhood leukemia, adult leukemia, and adult brain cancer (for a detailed comparison see O’Carroll and Henshaw, 2008). Where evidence is too disparate to readily support meta-analysis, because of, for example, a lack of a well-defined common ELF-EMF exposure metric, then the statistical aggregation used by CDHS, but not by IARC in their 2002 reviews, can be useful in supporting precautionary policies that could address the more common adult cancers, in addition to the relatively rare childhood leukemias.

#### Example of glyphosate

In analyzing the glyphosate case study, where IARC classified it as “probably” carcinogenic to humans, whilst the EU EFSA, the European Chemicals Agency (ECHA), and the U.S. EPA classified it as not carcinogenic, the workshop noted that the main reasons for such divergent evaluations of the same 2015 evidence did *not* include the different mandates of hazard and risk assessment, nor the difference between evidence for glyphosate alone and glyphosate within pesticide formulations. Divergences turned more on the evaluation of the animal evidence, where, for example, both U.S. EPA and EFSA describe a lack of significant pairwise comparisons as one reason for discarding positive findings due to positive trend analyses. This is in direct conflict with U.S. EPA Cancer Guidelines, which make it clear that a positive finding in *either* pairwise comparisons *or* trend tests should be sufficient to rule out chance: "Trend tests and pairwise comparison tests are the recommended tests for determining whether chance, rather than a treatment-related effect, is a plausible explanation for an apparent increase in tumor incidence… Significance in either kind of test is sufficient to reject the hypothesis that chance accounts for the result" [21]. The net effect of requiring both tests to be positive is an increase in the probability of a false negative outcome. U.S. EPA also noted that a lack of monotonic dose–response was a factor in its evaluation, and this was also used by EFSA to eliminate evidence of cancer. “The net effect of requiring monotonic dose–response is a severe reduction in the ability to detect a positive trend and a large increase in the probability of a false negative finding” [22]*.*

Other reasons why the EFSA/ECHA and U.S. EPA results diverged from IARC’s glyphosate evaluation included: limited analyses of the pre-neoplastic, or related non-neoplastic lesions; failure to evaluate support in the scientific literature for any of the tumors, relying entirely on the cancer bioassay results in drawing conclusions; use of study summaries or of studies that are not publicly available, in contrast with IARC’s exclusive use of publicly available reports, which promotes transparency; and use of historical controls, in contrast to IARC’s view that “it is generally not appropriate to discount a tumour response that is significantly increased compared with concurrent controls by arguing that it falls within the range of historical controls” [23]*.*

As with many other evaluations of evidence, the regulatory agencies put too much weight on the lack of consistency in study results. But, consistency is not to be expected in the raw tumor counts from studies done in different laboratories, at different times, using different diets, different exposure lengths, and different sub-strains of animals. U.S. EPA’s Science Advisory Panel, in their review of EPA’s draft risk assessment of glyphosate, recommended EPA do a pooled analysis to determine an overall effect, as IARC did. A subsequent pooled analysis adjusted for study differences demonstrated consistency for many of the tumors [22].

EU and U.S. Agencies also missed many of the tumors due to a failure to analyze all of the data using trend tests, relying instead upon the results of the analyses presented in the study reports rather than conducting its own thorough re-analysis of the data. Both EU Agencies and IARC differed over whether glyphosate was genotoxic or could induce oxidative stress with IARC identifying these as relevant mechanisms of action [24]. Finally, there appeared to be significant conflicts of interest involved in some of the evaluations with some scientists refusing to disclose conflicts of interest for the EFSA evaluation [25].

It is remarkable that despite glyphosate being the most widely used pesticide globally, roughly 1.8 billion pounds annually for both agriculture and non-agriculture uses in 2014 [26], at the time of the 2015 evaluations there was only limited monitoring of glyphosate in waterways and foods, which illustrates the need for more publicly available exposure information on environmental contaminants generally, and especially those released into the environment at high volumes.

#### Example of BPA

The case study on BPA [27] reinforces the PFOA conclusions about the importance of considering assumptions, argumentation, paradigms, and core beliefs when evaluating divergent evidence. Differences emerged about issues of methodology e.g., academic studies versus guideline studies (which are conducted by the product sponsor according to pre-set test methods, and submitted to regulatory agencies for the purposes of gaining product approval); about different disciplinary perspectives e.g., toxicology versus endocrinology; about linear or non-linear dose response curves; and about the reasoning regarding causality adopted by different evaluators. These divergences were explored in the government-academic-industry collaborative study, Consortium Linking Academic and Regulatory Insights on BPA Toxicity (CLARITY-BPA). The collaboration explored differences in the analysis and interpretation of study results among academic, government, and industry scientists [28, 29].

A possible approach to addressing these divergences would consider multiple types of uncertainty and would embrace the temporary and fragmented nature of evidence. This may help construct a more precautionary model of governance, in which uncertainties are not seen as a roadblock but more of a ‘speed bump’, to be addressed alongside quantitative evidence. A prudent approach to BPA governance may also include regulating all bisphenols, not just BPA, reducing their usage as much as possible, and investing in the development of inherently safe by design alternative substances and materials.

Substantial heterogeneity of scientists’ judgments about the quality of epidemiological studies has also been evident in the BPA case study even if the same criteria were used for the assessment [30]. However, this heterogeneity is not usually visible in reports produced under the collective signature of all the scientists involved. “Flattening heterogeneity” in this way can be a problem when it is not the result of true scientific agreement but only a secondary effect of consensus-based working procedures of agencies that experts have to follow.

The above case studies elevate myriad reasons for divergent outcomes of evaluations, which left unaddressed fail to lead to health-protective actions. Strategies to overcome these barriers must, therefore, at a minimum include approaches that: 1) make better use of existing data and information, 2) ensure timeliness, 3) increase transparency, consistency and minimize bias in evidence evaluations and 4) minimize the influence of financial conflicts of interest.

### Strategies to overcome barriers to protective actions: make better use of existing data and information

#### Include local knowledge and engage community members

If research outcomes are to address the most pressing problems for Environmental Justice communities, workers, and others most in harm’s way, it is critical to include meaningful participation by affected communities during research and decision-making processes [31]. This can include community representatives, local health care workers, local schools and parents, community organizations, local businesses, local unions and labor representatives, and others. For example, when trying to describe the potential impact of a pollution source or contaminated site, failing to include local knowledge from community members and others may bias the research results and limit the applicability of policy outcomes [32]. Information can be gathered using methods like community health surveys, community science and community scientists, community forums, and direct two-way dialogue between communities, scientists, and others. This information can inform the research design, data collection, and interpretation of the results. In addition, input and guidance from stakeholders and community members will help shape policy outcomes that are meaningful and address local concerns [33].

Early and ongoing public engagement with fenceline and other affected communities could help avoid repeating the practices that have failed disproportionately impacted communities for so long. In contrast, failing to include the data and knowledge of community residents, local first responders, schools and school nurses, local health care providers and others, limits the accuracy and relevance of the research findings and policy outcomes. Making better use of this information, as appropriate, by government agencies and others can advance research, reduce costs of data collection, address community concerns, and help fill in data gaps and uncertainties [34].

#### Include information on cumulative exposures and stressors

The failure of many risk evaluations to incorporate the cumulative impacts of overlapping environmental and social threats including systemic racism and poverty remains a serious limitation. Focusing narrowly on the risk of harm from a single facility without accounting for exposures from additional polluting facilities nearby will understate the potential harm from even a worst-case release. Additionally, failure to consider the unique characteristics of the surrounding population, including current and past exposures and social disadvantage will perpetuate disproportionate impacts in communities that are in most need of protective policies and practices.

While there are cases when truly independent committee members take divergent views on the degree of certainty needed to attribute causality of an adverse effect to a single chemical, these divergent views may be more likely where there are multiple chemical exposures. This is because the risk evaluation goes beyond typical single agent evaluations, and methods for combining effects can be challenging [35].

In one approach, researchers used publicly available facility self-reported air emissions data from the U.S. EPA Toxics Release Inventory (TRI) to identify counties which had reported air emissions of formaldehyde, a leukemogen and respiratory carcinogen, and additional chemicals linked to respiratory cancer in the U.S. EPA Integrated Risk Information System (IRIS) chemical hazard database. The analysis identified 19 counties with a cumulative total of 10 or more respiratory carcinogens, including formaldehyde, according to industrial facilities air emissions reports. Demographic analyses revealed correlations between the number of facilities emitting formaldehyde and living in a single-parent household, speaking English “less than well,” living in multi-unit housing, being disabled, or living in a mobile home [5]. Thus, as these communities shoulder disproportionately high levels of exposure to hazardous agents from multiple pollution sources and through numerous exposure pathways, combined with exposure to non-chemical stressors including poverty and linguistic isolation that further exacerbate the health risk and effects posed by hazardous agents – methodological advances are needed to risk evaluations and regulatory policies that capture and address the combined impact of these stressors.

#### Use cumulative impacts or burdens analysis to address disproportionate cumulative impacts of polluting and hazardous facilities

One approach to conducting cumulative evaluations is to identify the cumulative impacts or burdens. An example is a groundbreaking new law in New Jersey, U.S. (S.232 enacted in September 2020) imposed on industrial facilities that are applying for new permits, or to renew or expand existing permits. The new law requires an analysis of the cumulative environmental and public health impacts caused by a proposed activity in conjunction with existing stressors, when there is a specific facility permit application in a community that meets pre-defined socio-demographic thresholds [36]. If the community is disproportionately impacted, then the permit can be denied or can have conditions placed on it (depending on whether it is a new permit or a renewal/expansion). Public hearings and other community engagement requirements are integrated throughout the process. A limitation of the law is that it can only be triggered when facilities apply for new permits, renew permits, or seek to expand existing permits, leaving the status quo unexamined until the permit comes up for renewal or the operator seeks to modify the permit based on a change in the facility’s operation. Nonetheless, it provides a model for consideration by communities, stakeholders, and legislators wishing to address disproportionate cumulative impacts of polluting and hazardous facilities. A burden analysis can help fill the gap where there is not enough information to conduct a risk analysis, or where a risk analysis sets an unreasonably high technical or evidentiary bar that communities cannot meet.

#### Ensure comprehensive collection of data about environmental releases of all toxic pollutants and about population characteristics that identify population vulnerabilities

Regulatory agencies should be encouraged to fund and develop or upgrade existing public online tools to compile nationally consistent, robust, and reliable data for identifying overburdened communities, and create detailed visualization tools for hazardous exposures and other factors that increase a population’s vulnerability to environmental pollutants. These visualizations are only as good as the data that go into them, however. For example, although the U.S. EPA TRI requires U.S. facilities to report annually how much of certain toxic chemicals that may pose a threat to human health and the environment are released to the environment and/or managed through recycling, energy recovery and treatment [37], TRI data cover fewer than 800 chemicals, not all facilities are covered, there are high reporting thresholds, and the data are self-reported based on estimates. Without more comprehensive collection of data about environmental releases of all toxic pollutants, and modifications to ensure the reporting is reliable and accurate, these mapping tools will significantly understate the problem, that will bias the health effects of these pollutants toward the null finding in any investigation.

One example of a comprehensive public online mapping tool is CalEnviroScreen, developed by the California EPA, Office of Environmental Health Hazard Assessment (OEHHA) [38]. CalEnviroScreen incorporates 13 pollution burden indicators and eight indicators of population characteristics that identify population vulnerabilities relative to the effects of pollution exposure. Indicators used in CalEnviroScreen are regularly updated and added in direct consultation with impacted communities to meet their needs and realistically address exposures. An example of the use of mapping tools to benefit vulnerable communities is California Senate Bill 535 (2016) that requires 25% of the proceeds from the Greenhouse Gas Reduction Fund goes to projects that benefit disadvantaged communities. The California EPA used results from CalEnviroScreen to identify disadvantaged communities for investment [39]. U.S. EPA’s EJSCREEN includes 19 indicators, 12 environmental and seven demographic and is a step in the right direction for a nationwide mapping tool [40]. However, there are several limitations of EJSCREEN including that it omits important environmental indicators including drinking-water quality and indoor air quality, which EPA states is due to a lack of resources to collect underlying data, and there is uncertainty around demographic estimates, as they are derived from surveys, not a full census of all households [41].

#### Use toxicity studies even when they are not conducted for regulatory purposes

Risk assessors routinely disregard data from rodent bioassays that were not conducted according to methods described in pre-set test guidelines such as OECD Guidelines for Testing of Chemicals. Both the Organisation for Economic Co-operation and Development (OECD) Test Guidelines and the GreenScreen® For Safer Chemicals method down-grade injection studies, where a test substance was administered by subcutaneous or intraperitoneal injection. The OECD Test Guideline 478 Administration of Doses, states: “Intraperitoneal injection is not normally recommended unless scientifically justified since it is not usually a physiologically relevant route of human exposure” [42]. Similarly, the 2018 updated GreenScreen Guidance states that a study can be considered of “low confidence” if it uses an injection route of exposure [43]. Injection studies are routinely excluded from evaluations by the U.S. EPA Office of Pesticide Programs [44]. Discarding injection studies unnecessarily limits the final data set. For example, injection studies are useful to understand the mechanism of action of a test compound; pharmacokinetic models can account for the difference in route of exposure between injection and other exposure methods.

Other reasons given for excluding toxicity studies include that the study did not conform with OECD Principles of Good Laboratory Practices (GLP) [45–47]. The limitation of GLP requirements is that they are only meant to help ensure that the conduct of a study is properly documented. GLP requirements are not meant to provide any assurances that studies will answer meaningful and relevant questions, or that study protocols are appropriate or sufficiently sensitive to detect an adverse effect or outcome of treatment. In many cases, studies that are GLP-compliant may not address the most sensitive endpoints of concern. For example, this was demonstrated in the CLARITY-BPA collaboration, which reported that academic studies were more reliable at detecting low dose effects of BPA exposures, compared with regulatory Guideline studies [28].

Test Guidelines and GLP are requirements of industry test labs that conduct studies for the purposes of gaining regulatory approval. However, criteria that exclude or down-grade studies that are not GLP or Guideline-compliant will selectively bias against academic research. Since studies sponsored by industry – including regulatory Guideline studies – are more likely to report results that are favorable to the sponsor than those without industry sponsorship [22, 48–51], regulatory decisions that are overly-reliant on these data are likely to result in less or no regulation, or to drive increased demand, production and sale of the chemicals under scrutiny [52–55]. Instead, all studies should be used appropriately and with expert judgment.

As discussed later in this paper, the use of appropriate systematic review can provide transparent, explicit, standardized processes and methods for interpreting and integrating diverse streams of publicly available evidence into risk evaluations.

#### Use animal bioassays

Well-designed and well-conducted experimental animal studies of sufficient statistical power still represent one of the most predictive sources of evidence for primary prevention of disease from a hazardous agent [56]. There are many examples where the evidence of cancer, reproductive, neurologic, genotoxic, immunologic or other negative health impacts from animal studies preceded human evidence by many years, even decades (see Table 1) [57].

**Table 1:**
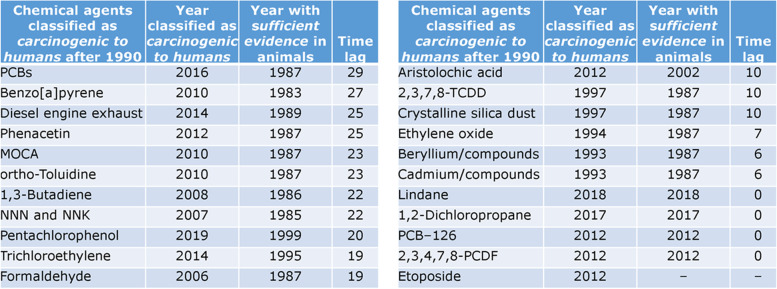
The year when sufficient evidence of carcinogenicity of a chemical agent was established in animal studies and the year it was classified as carcinogenic in humans, as reported in Monographs of the International Agency for Research on Cancer (IARC). (Table from presentation by V. Cogliano to the U.S. National Academy of Sciences, December 2021)

Some standard rodent bioassays may underestimate risk to humans, particularly during certain windows of susceptibility. For example, the OECD standard 2-year rodent bioassays start the test treatment around 8 weeks of age (young adult animals), whereas beginning the treatment from prenatal development has been shown to be a determinant factor in predicting more accurately the carcinogenic potential of chemicals, which often elicit their most detrimental effects during development [58–61]. A U.S. National Academies Committee noted that, “in general, estimates based on animal extrapolations have been found to be generally concordant with those based on epidemiologic studies…. and in several cases human data have indicated that animal-based estimates were not conservative for the population as a whole” [62]. Adjustments can be made to avoid a policy decision that may fail to adequately protect against potential harm, such as using additional uncertainty factors [63].

Information from animal models can be integrated with additional streams of information, as available, using systematic review methods that are consistent with established best science for evidence synthesis, as discussed in Adopt systematic review frameworks Section.

#### Use mechanistic data and key characteristics, to overcome data gaps and strengthen evaluations

Information on mechanism of toxicity, or key mechanistic processes that lead to the development of adverse health outcomes, is useful to strengthen or support an evaluation. Scientists have identified ten Key Characteristics (KCs) that reflect the properties of a cancer-causing agent: is electrophilic; is genotoxic; alters DNA repair; induces epigenetic alterations; induces oxidative stress; induces chronic inflammation; is immunosuppressive; modulates receptor-mediated effects; causes cell immortalization; alters cell proliferation, death, or nutrient supply [64–67]. These key characteristics of carcinogens have been applied in the evaluation of more than 70 diverse carcinogens at IARC [65] and are now used as the basis for the evaluation of mechanistic data by the IARC Monographs [23, 68]. For example, the IARC Preamble considers that “sufficient” evidence of cancer in experimental animals can support a cancer classification of “possibly” carcinogenic to humans, Group 2B [68]. However, when sufficient evidence in experimental animals is supported by “strong evidence in exposed humans that the agent exhibits key characteristics of carcinogens “the agent can be upgraded to Group 1, “carcinogenic to humans.” For example, this was done by IARC in 1997 for 2,3,7,8-TCDD, where mechanistic and animal data together supported the limited human data [69]. Where there is limited human evidence of cancer then “strong evidence in experimental systems that the agent exhibits key characteristics of carcinogens” can be used to justify a classification of a Group 2A, a “probable” carcinogen. Thus, mechanistic data can be pivotal, along with animal data, when human data are less than sufficient. A 2021 National Academy of Sciences (NAS) report on the IRIS program *Review of U.S. EPA’s ORD Staff Handbook for Developing IRIS Assessments,* recommended that “When available, KCs should be used to search for and organize mechanistic data, identify data gaps, and evaluate biological plausibility” [70].

Overall, much progress has been made in advancing approaches based on the key characteristics of carcinogens and other toxicants, including reproductive toxicants, endocrine disruptors, neurotoxicants, cardiotoxicants and hepatotoxicants to assemble and evaluate mechanistic data to support hazard conclusions [23, 64, 71–75].

#### Use “new approach methods”, including in silico, cell-based, and high throughput methods to support evaluations and up-grade hazard classifications

The last decade has seen an exponential increase in the development of computational, biological, and chemical tools promising to increase both the pace and the number of hazard evaluations, while reducing costs and the use of experimental animals. Both the EU and U.S. are heavily invested in applying “New Approach Methods” (NAMs) to regulatory decision-making, engaging partners that include government regulatory agencies, academic institutes, private for-profit entities such as methods development companies, and chemical companies – see for examples the websites for PrecisionTox and the U.S. EPA Collaborative Agreements for Computational Toxicology Research [76, 77].

Whilst these tools have great potential to provide useful information, there are a number of serious limitations that prevent NAMs from reliably and accurately identifying all chemicals with toxicity. These limitations include lack of biological coverage for complex developmental processes such as neurodevelopment, human genetic diversity, growth processes, and metabolic activity. Thus, complex and multi-system effects such as chronic and systemic health endpoints like developmental neurotoxicity, immunotoxicity, and endocrine effects may be missed with NAMs tests [78–80]. For this reason, expedited timelines to replace mammalian tests with high-throughput assays that are not capable of providing necessary information about health endpoints of critical concern, particularly for highly exposed and/or susceptible populations like workers, frontline community members, children, and pregnant women, would not be consistent with providing health protections for these populations.

The U.S. EPA Children’s Health Protection Advisory Committee (CHPAC) warned in a recent report against relying on high-throughput methods to downgrade hazard evaluations: “cell-based assays and other high-throughput toxicity tests, often called New Approach Methods (NAMs), have the potential to provide needed data and could be used to establish potential hazards or upgrade overall hazard identification. However, due to important limitations, data from NAMs cannot be used to rule-out a specific hazard” [81]. Similarly, government scientists recommended that: “when prioritizing chemicals for further study for a particular biological outcome … positive results (i.e., results that indicate potential harm) in relevant bioassays could be used to identify chemicals of concern, whereas negative results (i.e., results that indicate a lack of potential harm) are not sufficient to conclude a lack of concern given the limitations of current in vitro methods to simulate in vivo metabolism or predict effects in different tissues and across different life stages” [79].

To address these concerns, the overall framework of evidence integration should ensure that a hazard classification is not weakened based on speculative or limited data. Instead, results from NAMs should provide “actionable evidence,” that is, a scientific basis for health-protective actions. This could include: facilitating dose–response assessments to support regulatory standards; investigating the impact of complex chemical mixtures; identifying susceptible populations and quantifying differences in risk; investigating risks of complex chemical and non-chemical exposures. A committee of the U.S. National Academies of Sciences, Engineering, and Medicine is being convened to develop science-based recommendations for using NAMs in human health risk assessment, with a report expected in early 2023 [82].

#### Use data to provide real-world exposure information from human biomonitoring to support protective actions

Human biomonitoring (HBM) uses biomarkers within the body as an indicator of exposure, effect, susceptibility, or clinical disease. Biomarkers of exposure are measured in urine, blood, saliva, body fat, breast milk, and other body tissues. Thus, for example, Alghamdi et al. measured the polycyclic aromatic hydrocarbons (PAH) metabolites, 1-hydroxypyrene and hydroxyphenanthrenes in the urine of schoolchildren living near a refinery and found quantitative relationships to airborne PAH exposure [83]. Blood lead has been widely used as a biomarker of recent exposure to the metal and its compounds. In another example of exposure biomonitoring, deciduous baby teeth have been used to measure prenatal exposure to lead among poor communities of color located in close proximity to a lead-acid battery smelter in Los Angeles, U.S. [84]. Biomarkers of exposure can provide evidence of direct internal exposure in individuals. Biomarkers of effect, also measured in media such as blood or urine, are generally the products of metabolic processes and reflect the outcome of a potentially harmful process. Thus 8-hydroxy-2’-deoxyguanosine (8-OHdG) is an oxidation product of lipids of cellular membranes, proteins, and DNA, and is used as a biomarker of oxidative stress and carcinogenesis [85]. Delta-aminolevulinic acid dehydratase is an enzyme that is inhibited by lead and has been widely used as an indicator of effects of lead exposure. In another example, measuring changes in blood cholinesterase among those working with organophosphate and carbamate pesticides can be used as evidence of pesticide exposure, as well as providing medical confirmation of poisoning [86]. Combined with health-based risk assessments [87], HBM data can provide support for protective actions, build the basis for risk assessment decisions, and be used to evaluate the effectiveness of mitigation measures and policy interventions [88]. These are among the objectives of Biomonitoring California, a joint program of the California Department of Public Health, OEHHA, and the California Department of Toxic Substances Control (DTSC) created to measure the presence of toxic chemicals in California residents. Similarly, the European initiative for human biomonitoring project, called “HBM4EU,” is a coordinated effort across EU countries, the EEA, and the European Commission (EC). The project generates publicly available data on human internal exposures to chemicals, and the related effort to identify substances for human biomonitoring under the Partnership for the Risk Assessment of Chemicals (PARC) [88, 89].

HBM information can help shape public health and environmental policies, show whether chemical exposures are increasing or decreasing, identify groups of people who are more exposed than others, and evaluate the effectiveness of environmental protection programs [90]. However, while biomonitoring is evidence of exposure, it cannot necessarily identify the source of the exposure, so environmental exposure information is usually still needed. Moreover, monitoring on its own will not lead to reductions in exposure, and the use of HBM is too late to benefit people already exposed to unsafe levels of harmful agents. When production volumes are high for chemicals, human (or environmental) exposures are very likely occurring and calls for additional biomonitoring to verify these exposures are occurring may be used as excuses to delay action.

### Strategies to overcome barriers to protective actions: ensure timeliness

#### Use provisional values to deliver timely protections

Provisional toxicity values (which are used to set a dose/level of exposure of exposure) and default adjustment factors can be used to provide a measure of protection, when available chemical-specific data are inadequate for generating risk estimates that address the complex factors and stressors in vulnerable populations such as overburdened communities. A seminal report of the U.S. National Academies warned that the standard uncertainty factors – a 10X for interspecies differences when risk estimates are derived from an animal study, and a 10X for intraspecies differences across human populations, for a total of 100X – are likely to be insufficient to account for the real-world exposures experienced by vulnerable populations to multiple chemical and non-chemical stressors, [91]. In addition, the same committee described as a problem the implicit treatment of data gaps, or the absence of evidence of harm, as if it were evidence of the absence of harm [62]. Thus, risk-assessment policy should strive for plausible conservatism in the choice of default options to provide adequate health protections, particularly to vulnerable populations [62].

Regulatory agencies like the U.S. EPA, could increase the default adjustment factor for intra-species variability to a minimum of 42X, unless there are robust chemical-specific data to the contrary. This recommendation is supported by the estimate of human variability by the International Programme on Chemical Safety (IPCS), which relied on high-quality Toxicokinetic (TK) and Toxicodynamic (TD) data, primarily focused on healthy adults [92, 93]. Thus, as the 42X recommendation reflects differences only among adults and not differences across age/life stage of development, we also recommend the use of an additional adjustment factor for age/life-stage differences, which is currently required by U.S Congress for addressing additional susceptibility for pregnant women and children exposed to food-use pesticides. An additional factor, usually 3X or 10X (the Food Quality Protection Act safety factor), is incorporated into these risk assessments. Such an approach is underpinned by evidence that demonstrates that there are age-specific differences that must be accounted for [94]. Additionally, we recommend development of a separate default factor to account for exposure to multiple chemical and non-chemical stressors [95, 96]. This factor could account for human variability in susceptible subgroups. Risk assessments should include standardized approaches to include explicit descriptions of susceptible subgroups and the analysis of data sets that represent the multiple sources of variability within susceptible subgroups. Finally, based on NAS recommendations [91, 97], it is also critical to account for human variability in cancer dose–response analysis. Current cancer dose–response methods incorporate the estimated response at the median of the population. The NAS,however, recommends a default assumption of a 25-fold difference in cancer risk between the 95th percentile and the median human response.

#### Document uncertainties and data gaps, but do not let them delay protections

Although scientists tend to accept some amount of uncertainty as an inherent feature of any evaluation, informing the public about uncertainties may diminish trust and credibility as laypersons may attribute this uncertainty to a lack of professional expertise [98]. Further, in the public arena, scientific uncertainty is often exaggerated and even weaponized to cause distrust in science. This is detailed in David Michaels’ 2020 book, The Triumph of Doubt: Dark Money and the Science of Deception, and summated in a review of the book published in Nature magazine: “The principles of scientific inquiry involve testing a hypothesis by exploring uncertainty around it until there is a sufficient weight of evidence to reach a reasonable conclusion. Proof can be much longer in coming, and consensus still longer. The product-defense industry subverts these principles, weaponizing the uncertainty inherent in the process. Its tricks include stressing dissent where little remains, cherry-picking data, reanalysing results to reach different conclusions and hiring people prepared to rig methodologies to produce funders’ desired results” [99]. Resulting delays in adopting health-protective policies and practices [98–101] perpetuate health disparities and upholds inequitable systems [54]. Approaches are needed to integrate differing levels of evidence into decisions that must also consider human rights, environmental justice, feasibility, and benefits [102, 103]. Many prominent statisticians have raised concerns with over-reliance on statistical significance to disregard evidence of harm, instead recommending that statisticians and others “embrace uncertainty” rather than be held back by it [104]. Expert judgement can help interpret the impact on study design, data quality, and understanding of underlying mechanisms, which are “often more important than statistical measures” in determining causal relationships [104]. Importantly, when feasible, the uncertainty on the magnitude and direction of the effect should be documented. That is, document whether the absence of information is more likely to over-estimate or under-estimate harm. This can be useful in considering the addition of numerical adjustment factors to provide a margin of protection around a risk estimate and supporting health-protective policies and practices.

#### Evaluate chemicals based on hazard

Taking protective action on chemicals based on their inherent hazardous properties, rather than on a risk-based approach, is much less data intensive, as it does not require as much information about the exposure to the chemical. This approach can inform regulators and others about prioritizing chemicals of concern for future risk assessments, as well as encourage protective policies and practices that can be implemented immediately to mitigate or eradicate exposures to protect the most impacted populations and communities against chemicals of concern.

One model is the California Safer Consumer Products (SCP) regulations, which require DTSC to only demonstrate a *potential* for exposures and significant or widespread adverse impacts before health-protective actions is taken [105]. This hazard-based approach is designed to support regulations of product-chemical combinations of concern in the face of limited information to protect vulnerable human populations, threatened and endangered species, sensitive habitats, and impaired environments [105]. The SCP regulations authorize DTSC to designate Priority Products, which are specific consumer products (excluding pesticides, food, pharmaceuticals, and medical devices) that contain one or more chemicals that appears on one or more established authoritative lists referenced in the SCP regulations. To identify Priority Products, DTSC does not need to conduct a formal risk assessment, nor a weight-of-evidence analysis. It only needs to find that exposure to a chemical of concern in the product has the potential to “contribute to or cause significant or widespread adverse impacts” to human health or the environment [105]. One reliable study alone that indicates such potential can suffice for DTSC to act and regulate a product-chemical of concern as a Priority Product. The formal identification of a Priority Product requires the responsible entities (often the product manufacturers) to either remove the chemical or product from the California market or conduct an Alternatives Analysis to evaluate whether the chemical(s) in question can be replaced with a safer alternative [106]. Depending on the results of the Alternatives Analysis, DTSC may then consider regulatory responses to protect public health and the environment, including disclosure of chemical use to consumers, limits on use, or sales bans. An example of a rulemaking under the SCP regulations is the listing of carpets and rugs containing per- and polyfluoroalkyl substances (PFASs) as a Priority Product.

#### Evaluate chemical classes

Rather than evaluating chemicals one at a time, it can save both time and resources to evaluate numerous chemicals together as a class, treating those with little or no data on hazard or exposure as if they are similar to those chemicals in the class for which there is more data. A class approach is needed because, for most chemical classes, information on toxicity and other hazardous properties is only available for a small number of members of the class, thus leading to delays in evaluation and regulation for the data-poor chemicals. Evaluating and regulating entire chemical classes based on information on a few members of the class and common properties shared by all members of the class is one of the most effective ways to ensure timely regulations, and to prevent regrettable substitutions such as replacing Bisphenol A with Bisphenol S which is not yet restricted but shares a similar toxicity profile. IARC also uses mechanistic evidence as a basis for identifying whether an agent belongs to a class for which other members have already been linked to cancer.

As an example, only a very small percentage of the roughly nine thousand PFASs have publicly available toxicological information from epidemiologic, animal, or in vitro studies [107]. This class includes perfluoroalkyl acids, perfluoroalkylether acids, and their precursors; fluoropolymers and perfluoropolyethers; and other PFASs. However, all PFASs share one common characteristic—they are either highly persistent “forever chemicals” themselves or degrade into other highly persistent members of the PFAS class [108]. Highly persistent chemicals accumulate in the environment, eventually exceeding the thresholds for known and as yet unknown adverse impacts, and are difficult to remove from the environment. Regulating PFASs as a chemical class would prevent a regulated PFAS from being replaced with another PFAS that is not yet regulated.

Many well-known historical chemical pollution problems were the result of the release of highly persistent chemicals, such as polychlorinated biphenyls (PCBs) and chlorofluorocarbons (CFCs). Consequently, persistence was adopted by the 2001 Stockholm Convention on Persistent Organic Pollutants and by the EU Registration, Evaluation, Authorisation, and Restriction of Chemicals (REACH) regulations in 2007 as a hazard criterion for the better management of chemicals, with “very persistent” being a cause for identifying “chemicals of serious concern.” In July 2021, California DTSC became the first regulatory agency to use persistence as the basis for regulating PFASs as a class in certain consumer products (carpets and rugs) [109]. Regulation of highly persistent chemicals, for example by restriction of emissions, would not only be precautionary, but would serve to prevent poorly reversible future impacts [110].

Importantly, IARC also uses mechanistic evidence as a basis for identifying whether an agent belongs to a class for which other members have already been linked to cancer.

#### Implement product labeling and public right-to-know laws

Public disclosures such as warning labels on consumer products are helpful to inform consumer purchasing choices, and function to encourage manufacturers to steer away from harmful ingredients that would trigger the need for a warning label [111]. The California Proposition 65 law (Prop 65), for example, requires the State to publish a list of chemicals known to cause cancer, birth defects or other reproductive harm and requires businesses to provide public warnings about significant exposures to those chemicals. The Prop 65 website notes instances where the law has resulted in public health protections, including: removal of the solvent trichloroethylene (TCE), linked to both cancer and birth defects, from most correction fluids; removal from reformulated paint strippers of methylene chloride, linked to both cancer and death from asphyxiation; reductions in the lead content of glazes on ceramic tableware; and, removal of the toxic solvent toluene, a carcinogen, mutagen, and reproductive toxicant, from most nail polishes and other nail care products regularly handled by salon workers that are largely ethnic minority women of reproductive age [112]. Removal of these hazardous chemicals from products delivered health protections to both workers and consumers [113]. Prop 65 also drove California to lower permissible limits on toxic air emissions for ethylene oxide, hexavalent chromium, and chloroform [113].

In summary, requirements for warning labels on consumer products can be an effective means of helping consumers make informed purchasing choices, as well as disincentivizing the use of harmful ingredients [113]. The QR (quick response) code system, created in a standard format to be understandable to nonexpert readers could also be implemented in some cases to complement warning labels on the product, to direct the more engaged user to a website with additional details or translations into additional languages. Nonetheless, one should not assume that a product is safe – even when used according to the label – just because it can be easily purchased.

### Strategies to overcome barriers to protective actions: increase transparency and consistency, and minimize bias in evidence evaluations

There is a need for the organizations that conduct chemical evaluations of environmental exposures to adopt empirically-based tools and methods for the evaluation of evidence [114]. The current lack of an agreed upon method has resulted in a large degree of inconsistency across national and international agencies and organizations in the processes and methods used to conduct chemical evaluations, including how to identify, select, and evaluate the evidence [115]. The use of such heterogeneous methods is one cause of the many divergent evaluations of the evidence on the health effects of hazardous agents. This reduces the level of confidence the public has in the conclusions of the assessments made by these various organizations [115]. Divergent evaluations can also increase uncertainties about the evidence, which often leads to policy inaction.

#### Adopt systematic review frameworks

The evaluation and integration of evidence can be done with more consistency across regulatory agencies by relying on established guidelines that use standard processes and methods, and by applying systematic review methods that are consistent with established best science for evidence synthesis. Systematic reviews increase the transparency and objectivity of an evaluation of the evidence as they allow end users to identify how the questions were formulated, the searches of evidence conducted, and how the evidence used in the final recommendation was evaluated. These steps therefore reduce and limit bias in each part of the review process [116]. Importantly, when divergent evaluations of the same body of evidence are made, the reasons for such divergence can be readily identified. A recent analysis of the methodological strengths and weaknesses of a sample of “expert-based narrative” and “systematic” reviews in environmental health found systematic reviews produced more useful, valid, and transparent conclusions compared to non-systematic reviews [117].

Authoritative bodies and academic scientists developed and implemented several robust, reliable peer-reviewed systematic review methods. Notable ones include: the IARC Monographs Preamble 2019 [23, 68]; University of California San Francisco’s Navigation Guide (UCSF Navigation Guide) [118]; the U.S. National Toxicology Program’s Report on Carcinogens Handbook [119] and its Office of Health Assessment and Translation Systematic Review methodology (National Institute of Environmental Health Sciences [NIEHS] NTP-OHAT) [120]; the Systematic Review and Integrated Assessment of endocrine disrupting chemicals (SYRINA) [121]; and World Health Organization and International Labour Organization (WHO-ILO) systematic review methods to estimate the work-related burden of disease and injury [122]. These methods have been recognized by the U.S. NAS in multiple reports that recommend use of a robust, systematic, and transparent methodology to improve understanding of environmental health evidence, which will in turn support more timely and transparent decision-making [70, 123, 124].

Standard definitions and criteria for systematic review need to be adhered to by the organizations using them. The Institute of Medicine (now the National Academy of Medicine) defined systematic review as a “scientific investigation that focuses on a specific question and uses explicit, pre-specified scientific methods to identify, select, assess, and summarize the findings of similar but separate studies” [125]. The Institute of Medicine has well established standards for conducting a systematic review [125]. The term “systematic review” is being corrupted, however, because researchers and organizations are appropriating the term without adhering to the required systematic approach [126, 127]. For example, the systematic review method developed by the Texas Commission on Environmental Quality (TCEQ) and U.S. EPA as part of the implementation of the Frank R. Lautenberg Chemical Safety for the 21st Century Act (which amended the Toxic Substances Control Act [TSCA], the U.S.’s primary chemicals management law), and used to evaluate the first ten chemicals under the Act, fails to meet many of the standards of a well conducted systematic review [124, 125, 128]. The U.S. NAS recently comprehensively reviewed the “TSCA method,” developed under the Trump Administration, and identified that it “does not meet the criteria of ‘comprehensive, workable, objective, and transparent systematic review methods” [124]. The application of U.S. EPA’s TSCA method resulted in the exclusion of high-quality research from EPA’s decision-making, and may have therefore led to an underestimation of the true harms of these chemicals. U.S. EPA has announced that it would no longer use that method [129].

#### Conduct rapid reviews where needed

Systematic reviews are an effective tool for a rigorous evaluation of the evidence, however, when trying to address hazardous agents where there is an immediate exposure threat and time is limited, a systematic review may have to come after protective actions, if at all. There are newer approaches that can be adapted to accommodate the need for swifter action including the aforementioned hazard-based approach (Evaluate chemicals based on hazard Section) in which one needs only demonstrate a potential for exposures and significant or widespread adverse impacts before health-protective action is taken. When timely evaluations of the evidence are required, rapid review methods can be a valuable advance in the field of environmental health. Rapid reviews are a type of systematic evidence synthesis that omits certain methodological steps to accelerate the process of performing traditional systematic reviews of the evidentiary base when high-quality systematic reviews are not available. This approach helps to produce evidence syntheses in a timely manner that meets end-users’ needs. Rapid reviews have been conducted by Cochrane to support the development of evidence-based recommendations related to COVID-19, such as “What is the most effective screening strategy for COVID-19?”, within a rapid time frame (three to six months) [130]. Provisional rapid review methods recommendations have been developed by the Cochrane Rapid Reviews Methods Group that can be used to guide researchers in their implementation [131]. Rapid reviews should be used cautiously and, in many cases, may need to be followed by a standard systematic review to confirm the findings of the rapid review.

#### Use risk of bias tools

Risk of bias tools are intended to provide a consistent approach to the evaluation of a study’s design and conduct to determine if it may have introduced a systematic error in its results [132]. Well-designed risk of bias tools can be used to assess the internal validity of a study by providing a set of criteria and decision-rules to guide investigators in making qualitative judgements for each domain the tool assesses. Different tools with different domains of bias apply to different streams of evidence, such as epidemiologic, toxicologic, and mechanistic studies [133]. Epidemiologic and toxicologic risk of bias tools evaluate, for example, the validity of exposure and outcome assessment methods used in a study.

However, these tools have limitations. One of the key challenges is ensuring the risk of bias tools are focused on potentially important biases, without being overly prescriptive or so unstructured that expert judgements cannot be reported or validated [134]. In addition, advances are needed that assess the effects of potential biases on the direction and magnitude of effect. Further, risk of bias tools are needed that address not only studies on hazard and risk, but also prevalence of exposure to estimate burden of disease [135].

A recent study found that tools that use an overall risk of bias rating may reduce the available evidence to evaluate the health effects of chemical exposures by excluding studies based on only one methodological or reporting limitation, leading to an inaccurate conclusion [136, 137]. These findings are consistent with the 2021 NAS report on the IRIS Program, which found, based on data from recent IRIS assessments that used such a risk of bias approach, that the proportion of human studies excluded from further consideration ranged from 0 to 50 percent for human epidemiological studies, and 0 to 41.5 percent for animal studies [70]. Recognizing this concern, two separate 2021 NAS reports recommended that “study evaluation ratings should not be used to exclude studies” [70], “Do not exclude studies based on risk of bias, study quality, or reporting quality” and “Do not use numeric scores to evaluate studies; replace them with domain-based scoring as is done in the tools used in the Navigation Guide and OHAT” [124].

To avoid discarding valuable information, risk of bias assessments should be performed for each individual study, and the evidence base should then be assessed in its entirety. This allows an exploration of the potential effects of various biases. The 2021 NAS report makes this point: “While there is inevitably variation in the internal validity and risk of bias across individual studies, it is standard practice to include all studies, even the studies with a high risk of bias into the evidence synthesis… Once a study is determined to be eligible, the study could be included in the synthesis and the risk-of-bias assessment and its limitations accounted for in any qualitative or quantitative synthesis… In the synthesis step, low-quality studies may be excluded as a sensitivity analysis, but it is inappropriate to leave them out of synthesis completely” [124].

#### Leverage meta-analyses to support risk estimates

Meta-analyses, the statistical combination of results from two or more individual studies, may be informative to regulatory decision-making as they can increase confidence in a body of evidence and therefore the overall conclusion [117, 132]. Use of a meta-analysis can increase the precision of an effect estimate, by basing the estimate on a larger number of studies. Meta-analytical estimates can also be used to quantify effects across sufficiently homogeneous studies, provided the original studies have sufficient quantitative information. Meta-analyses frequently underpin Health Impact Assessments (HIA) and cost–benefit analyses (CBA) of interventions, such as policies to reduce air pollution. Meta-analyses allow for the possibility of conducting sensitivity analyses across studies. Sensitivity analyses are useful to identify how dependent the output is on particular input values, and thereby increase transparency and better inform the decision-making process. For example, sensitivity analyses can be used to explore heterogeneity, due to potential sources of bias such as financial conflicts of interest (COI) or of the influence of duration and life-stage timing of exposure on the study results. If inappropriate study designs are combined and within-study biases and reporting biases are not carefully considered and accounted for, they have the potential to be misleading and lead to erroneous conclusions being drawn about the evidence [132]. Sometimes, high quality individual studies may be more informative [138].

It is important to note that meta-analyses do not alleviate the need for critical review of all available data; thus, both the meta-analyses and the original research studies should be subjected to a rigorous critical review. Nonetheless, meta-analyses can provide important opportunities when synthesizing study results to strengthen hazard evaluations, and should be used as appropriate [117].

#### Use guidance documents and frameworks

Regulatory agencies often rely on frameworks and structured approaches for how science and technical information is evaluated and used to inform policies and regulations. Guidance documents are helpful by providing a generally accepted process for using available information, to move past data gaps and uncertainties to an evaluation and policy outcome. In this way, adhering to guidelines reduces process uncertainty and increases consistency, transparency, and accountability in the use of scientific information and in the policy outcome. Some examples of helpful guidance documents are the U.S. EPA Cancer Guidelines and its accompanying Supplemental Guidance for Assessing Susceptibility from Early-Life Exposure to Carcinogens, and the IARC Monographs Preamble 2019 [21, 23, 68, 139].

Guidance documents or frameworks that address how evidence is interpreted and integrated will be most useful if they are kept updated with advances in processes, methods, and best practices. Additionally, there is a need for guidance that accommodate policy decisions based on varying levels of certainty of the evidence. For example, the benefits of reduced exposure to a chemical with a “suggestive” relationship to serious heath endpoints, such as cancer, may be higher than the benefits of reduced exposure to a chemical with a deemed “known” relationship to less serious health endpoints. It may be therefore unfortunate to take account of the latter but not of the former [103]. In that case, suggestive evidence as characterized by cancer guidelines should be used as the basis for quantitative assessments of harms and should be the basis for policy decisions.

#### Develop evidence-to-decision frameworks for environmental health

Science on the harms of hazardous agents and the effectiveness of interventions to mitigate these harms is only one element of decision-making. Other considerations include equity across population groups, benefits, costs, feasibility, and the availability of alternatives. Guideline panels and other groups of experts can use evidence-to-decision (EtD) frameworks to provide a transparent and structured way to develop recommendations and inform decisions. Panels use these frameworks to consider explicit criteria individually and in aggregate, as they develop recommendations and decide on the relative merits of potential interventions [102].

To ensure that historically marginalized communities are not further subjected to health disparities, EtD frameworks in environmental health must consider key criteria that address health equity and environmental justice. A recent scoping review of existing EtD frameworks identified the need for improved approaches for decision-making in environmental health and recommended frameworks that integrate other factors into the decision making process beyond the benefits and harms of a proposed intervention, including health equity and human rights [102]. Such frameworks can help make meaningful, relevant, and actionable recommendations in cases of data gaps and uncertainty [102].

Considerations of who pays the costs of pollution reduction measures should also be included in policy decisions—the individual, civil society, or industries that may be the source of the harmful environmental exposures. These are value-laden considerations that should be informed by consultation with relevant stakeholders, such as health-impacted communities, health care workers, community representatives, consumer representatives, and others. Moreover, the claimed economic costs of pollution mitigation or intervention measures should be subjected to appropriately rigorous and transparent scrutiny [140]. These communities are often told that clean-up or other mitigations are not possible due to cost or feasibility issues, or because it may threaten the economic stability of their community [141–144]. Communities are not afforded the protections they deserve if the historical, institutional, cultural or behavioral systems that disadvantage and harm low-income and communities of color are not addressed [4, 6, 8]. Economic feasibility should not be used as an excuse to weaken policy, and disregard persistent environmental health disparities.

### Strategies to overcome barriers to protective actions: minimize the influence of financial conflicts of interest

#### Identify and account for industry influence in the research process

As demonstrated by myriad well-characterized toxicants including lead, air pollutants, including greenhouse gases and tobacco smoke, those with financial a stake in the manufacture, distribution and sale of hazardous agents are incentivized to ignore, downplay, distort or create confusion around the early warning signs on the harms of their products, which leads to delay regulatory action to the detriment of public health [53, 54, 145, 146]. It has been demonstrated across pharmaceutical, tobacco, nutrition, chemical and ELF-EMF research that studies that have an industry sponsor or an author with a financial COI are more likely to produce results and conclusions that favor the sponsor’s product than studies without an industry sponsor or author with a COI [52, 147–151]. This bias remains even when we control for the other methodological risks of bias (or internal validity) that could influence a study’s results [52, 147, 150]. Industry sponsors or authors with a COI can intentionally bias the research process through various mechanisms, including how the research question is framed, through the design and conduct of a study; how the events are coded; how the study data are analyzed and the results and conclusions are reported [152–155]. For example, in a 2019 evaluation of data linking exposure to the herbicide paraquat with a potential risk of Parkinson’s Disease, the U.S. EPA Office of Pesticide Programs (EPA Pesticide Office) identified a distinct difference in reported outcomes based on study sponsorship [44]. EPA noted that industry-sponsored studies "mostly present null results using an exposure design similar to studies in the literature that report significant decline in dopaminergic neuron counts." Reviewing the same data set, U.S. National Toxicology Program scientists identified that the industry-sponsored study design made it unlikely to identify adverse outcomes, as the duration of the study was “too short and dosing too infrequent” to reliably cause observable adverse effects [44].

Companies that produce or manufacture chemicals, trade associations that may represent those companies, or authors who receive financial support from the chemical industry can be expected to gain financially from demonstrating the chemicals they are evaluating are safe for use in commerce. Financial incentives, therefore, may sway industry and industry-sponsored scientists to alter the research process and distortions in the interpretation of evidence to bias findings regarding the harms of the chemicals they evaluate. Such findings could be used to limit, delay, or obstruct regulation of these chemicals, or further market the benefits of these chemicals to drive demand, production, and sale. Therefore, the potential effect of industry influence in the research process must be accounted for when evaluating a body of evidence, which can only be achieved through 1) full disclosure of financial COI of a study and 2) the use of methodological approaches such as with risk of bias tools that consider industry sponsorship and author COI as a risk of bias to the validity of the study results discussed below in the Section Consider financial conflict of interest as a risk of bias so that manufactured doubt is not used to delay protective actions.

#### Strengthen science disclosure policies

An essential step in evaluating the potential influence of financial COI on research is for the public to be able to identify who funded the research and whether the study authors have financial COI, particularly with companies that manufacture, process, or distribute chemicals, or with any trade associations that may represent those companies [156]. Public disclosure of any potential COI “is necessary to protect the integrity of scientific discourse,” according to the 2020 Position Statement of the International Network for Epidemiology in Policy [157].

In a study examining the prevalence of financial COI disclosures in biomedical research in journals subject to the International Committee of Medical Journal Editors (ICMJE) policies, only approximately 23% of articles conformed to ICMJE disclosure standards and included a COI disclosure [158]. The implementation of policies on disclosure is the responsibility of the journals that publish environmental health research. These policies should extend beyond only the authors and include peer reviewers and journal editors that were involved in either the peer-review or decision-making processes. All financial interests over a well-defined period should be disclosed, including but not limited to grants, honoraria, employment, litigation support, and the promise of future financial support [159]. The Collegium Ramazzini has called on scientific journals to establish mechanisms consistent with international best practices that provide disciplinary action for editors, authors, and peer reviewers who fail to disclose financial conflicts and competing interests, noting that in the absence of effective implementation, policies mean little [160].

Cochrane’s policies on funding and author COI are a standard that environmental health journals could follow. Every author of a Cochrane review must fully disclose all COI according to ICMJE recommendations before they publish a protocol, review, or update of a review. Cochrane’s policy is that all reviews must have a majority of authors without a COI, and that the first author must have no conflicts [161]. Note that the IARC Monographs Programme goes further, requiring that working group members do not have any real or perceived COI. Cochrane reviews cannot be funded or commissioned by any industry sponsor that may have a vested interest in the reviews findings. If authors fail to disclose financial COI, punitive methods that can include banning the author from publishing in that journal or considering retraction of the article should be considered [159].

Policies to enforce the reporting of financial COI among individuals that serve on scientific committees are critical for transparency. An example of best practices is the *IARC Monographs* program, which uses strict COI standards coupled with an independent verification process. Prospective working group members complete WHO’s Declaration of Interests to report financial interests, employment and consulting, and individual and institutional research support. IARC generally does not invite experts with COI and places restrictions on the participation of the few, if any, who might have a COI [68]. When publishing *Monograph* findings in *The Lancet Oncology,* COI statements are independently summarized by the journal editor [162], further ensuring transparency and checks on the reporting and assessing of author disclosures.

#### Consider financial conflict of interest as a risk of bias

Government agencies and other organizations that conduct chemical evaluations use a variety of methods to assess the potential for bias in primary research studies, but often do not assess financial conflicts of interest [47, 163]. Assessing risk of bias—including funding source and author COI in the primary studies included in systematic reviews—is a critical component of a systematic review [136]. As there is rarely sufficient public documentation available, to determine if a study sponsor has deliberately introduced a bias in one or all of the steps of the research process, a practical approach to account for it is to consider sponsorship as a risk source of bias [52]. Importantly, including funding source and author COI as a risk of bias domain does not remove studies from the body of evidence, it only means evaluating its impact on the overall quality of a body of evidence. A U.S. National Academies committee recommended that, “Funding sources should be considered in the risk-of-bias assessment conducted for systematic reviews that are part of an [EPA] IRIS assessment” [164] and in their 2021 report on the IRIS Assessments that they “should describe how to detect and assess the effect of funding bias on the confidence of study ratings from evidence evaluation or effect estimates from synthesis” [70]. Consistent with NAS recommendations, current risk of bias tools need to include study sponsorship. Some organizations and methods, including UCSF Navigation Guide, assess both author COI and funding sources in human and animal studies in their risk of bias tool [118, 135, 165–167], as does the WHO-ILO joint project to assess work-related burden of disease and injury [168].

#### Increase funding for research in the public interest

In addition to a much-needed increase in public funding for environmental health research, funding mechanisms are needed whereby industry increases its contributions to the costs of toxicity testing, environmental monitoring, biomonitoring, and other research in the public interest. An example of this is in Italy where the testing of the safety and efficacy of drugs is funded from taxes paid by the pharmaceutical industry’s drug promotion [169]. The need for such a system has been identified by an international scientific society of 180 physicians and scientists from 35 countries, Collegium Ramazzini, in a public statement calling on “national and international official bodies to set up evaluation procedures that systematically orient funding towards research centers, researchers, and research activities with demonstrated commitment to competence and impartiality in assessing health effects” [160]. Recently updated laws governing chemical manufacturing and use in the U.S. and EU have attempted to shift the burden of toxicity testing onto manufacturers, with varying success – data gaps still abound, and in many cases the data received is of very poor quality. See for example the peer review report of the data submitted to both the EU and U.S programs for Pigment Violet 29 [170].

## Recommendations

We summarize the findings above into the following four key recommendations to minimize divergent evaluations of the evidence and to guide and inform the development of transparent, timely, reliable, and valid evaluations of evidence to support health-protective actions against hazardous agents:

### (1) Make better use of existing data and information

- Early and meaningful engagement with impacted populations to include local knowledge should include advocates and community members on committees and panels; their perspectives are critical. These perspectives can shape the research process and lead to policy outcomes that are meaningful and address local concerns. Importantly, they can help avoid repeating the practices that have failed disproportionately impacted communities for so long.
- It is important to incorporate the cumulative impacts of environmental and social threats, including systemic racism and poverty that can amplify the impact of hazardous agents.
- Comprehensive data on environmental releases of all toxic pollutants and the population characteristics that indicate vulnerabilities are needed to identify factors that contribute to health disparities, including risk factors that may vary by race/ethnicity or income and contribute to differential health outcomes.
- New or advanced methodologies should be used to strengthen risk evaluations and support health-protective regulatory and policy decisions.

### (2) Ensure timeliness

- Uncertainty and data gaps should not be used to delay needed protective measures.
- The approach taken to gather and synthesize evidence should consider factors such as urgency, available resources (people and financial), and the volume of available evidence.
- The strength of evidence needed to justify action is context specific and also depends on the plausible consequences of inaction.
- Evaluating and regulating entire chemical classes can facilitate timely protections and prevent regrettable substitutions of restricted chemicals with similar chemicals that are not yet restricted but similarly harmful.

### (3) Increase transparency and consistency, and minimize bias in evidence evaluations

- Systematic review methods should be utilized as appropriate, to increase transparency, minimize bias, and increase rigor in scientific evaluation and risk assessment.
- Guidelines and frameworks are needed that provide structured approaches for how science and technical information is evaluated and integrated into policies. To be relevant, they must be kept updated.
- The key paradigms, theoretical approaches, assumptions, values, choices, and judgments used in the evidence evaluations must be transparent so that the points of divergence across evaluations can be better understood.

### (4) Minimize the influence of financial conflicts of interest

- Full disclosure of financial COI is necessary but not sufficient to manage financial COI.
- Stricter disclosure policies should be enforced for research design and conduct, publication, peer review, and policy outcomes.
- Increased funding is needed for research in the public interest, from both public and private sources.
- Financial COI should be considered a risk of bias when evaluating primary studies.

## Conclusion

It is often a challenge to determine when a hazard or risk evaluation is “good enough” to support health-protective policies and actions, as data gaps and uncertainties likely persist. Early protective actions may necessarily rely on a less robust evidence base, giving scientists less confidence in the conclusions, but due to ongoing exposures it is important that these early scientific indicators be incorporated into decision making. In such cases, where a more limited assessment is conducted, it will be important to revisit the assessment as new information becomes available. Every adverse outcome that is unaddressed in a limited assessment is a potential disease that is not investigated, minimized, and compensated. The *Late Lessons* book showed via over 30 case studies that evidence of both exposures and harms emerged with more research, and exposure limits have in many cases been ratcheted down to more protective levels over time. Thus, there is good justification in taking early action when there is some signal of harm.

The four key recommendations identified in this paper provide a cornerstone for producing reliable evaluations that are applicable to various policy and regulatory settings. If properly implemented, they will support policymakers, politicians, and the public in taking timely, health-protective action to mitigate harms from hazardous agents.

## Data Availability

No new data was generated for this paper; only existing, publicly available data were used. DOIs and hyperlinks are included throughout the literature cited.

## Abbreviations

BPA: Bisphenol A
CBA: Cost benefit analyses
CDHS: California Department of Health Services
CFCs: Chlorofluorocarbon
CHPAC: U.S. EPA Children’s Health Protection Advisory Committee
CLARITY-BPA: Consortium Linking Academic and Regulatory Insights on BPA Toxicity
COI: Conflicts of Interest
DTSC: California Department of Toxic Substances Control
EEA: European Environment Agency
EC: European Commission
ECHA: European Chemicals Agency
EFSA: European Food and Safety Authority
EJ: Environmental Justice
ELF-EMF: Extremely Low Frequency – Electrical Magnetic Fields
EU: European Union
EtD: Evidence-to-decision
GLP: Good Laboratory Practices
U.S. EPA: United States Environmental Protection Agency
HBM: Human Biomonitoring
HBM4EU: European Initiative for Human Biomonitoring
HIA: Health Impact Assessments
IARC: International Agency for Research on Cancer
ICMJE: International Committee of Medical Journal Editors
ILO: International Labour Organization
IPCS: International Programme on Chemical Safety
IRIS: Integrated Risk Information System
KCs: Key Characteristics
NAMs: New Approach Methods
NAS: The National Academy of Sciences (U.S.)
NIEHS: National Institute of Environmental Health Sciences
NRDC: Natural Resources Defense Council
NTP: National Toxicology Program
OECD: Organisation for Economic Co-operation and Development
OEHHA: California EPA Office of Environmental Health Hazard Assessment
OHAT: Office of Health Assessment and Translation, U.S. National Toxicology Program
PAH: Polycyclic Aromatic Hydrocarbons
PARC: Partnership for the Risk Assessment of Chemicals
PCBs: Polychlorinated biphenyls
PFASs: Per- and polyfluoroalkyl substances
PFOA: Perfluorooctanoic acid
PRHE: Program on Reproductive Health and the Environment
REACH: Registration, Evaluation, Authorisation, and Restriction of Chemicals
RIVM: Dutch National Institute for Public Health and the Environment
SCP: California Safer Consumer Products
SYRINA: Systematic Review and Integrated Assessment of endocrine disrupting chemicals
TCE: Trichloroethylene
TRI: Toxics Release Inventory
TSCA: Toxic Substances Control Act
TCEQ: Texas Commission on Environmental Quality
TD: Toxicodynamic
TK: Toxicokinetic
UBA: German Umweltbundesamt
UCSF: University of California, San Francisco
UK: United Kingdom
U.S.: United States
WHO: World Health Organization
WHO-ILO: World Health Organization-International Labour Organization
